# Nutritional regulation of genome-wide association obesity genes in a tissue-dependent manner

**DOI:** 10.1186/1743-7075-9-65

**Published:** 2012-07-10

**Authors:** Piriya Yoganathan, Subashini Karunakaran, Maggie M Ho, Susanne M Clee

**Affiliations:** 1 Department of Cellular and Physiological Sciences, University of British Columbia, Vancouver, BC, Canada; 2 Life Sciences Institute, Department of Cellular and Physiological Sciences, University of British Columbia, 2350 Health Sciences Mall, Vancouver, BC, V6T 1Z3, Canada

**Keywords:** Obesity genes, Genome-wide association, Gene expression, High fat diet, Feeding and fasting, Gene-diet interaction, Adipose tissue, Brain

## Abstract

**Background:**

Genome-wide association studies (GWAS) have recently identified several new genetic variants associated with obesity. The majority of the variants are within introns or between genes, suggesting they affect gene expression, although it is not clear which of the nearby genes they affect. Understanding the regulation of these genes will be key to determining the role of these variants in the development of obesity and will provide support for a role of these genes in the development of obesity.

**Methods:**

We examined the expression of 19 GWAS obesity genes in the brain and specifically the hypothalamus, adipose tissue and liver of mice by real-time quantitative PCR. To determine whether these genes are nutritionally regulated, as may be expected for genes affecting obesity, we compared tissues from fasting and non-fasting animals and tissues from mice consuming a high fat high sucrose diet in comparison to standard rodent chow.

**Results:**

We found complex, tissue-dependent patterns of nutritional regulation of most of these genes. For example, *Bat2* expression was increased ~10-fold in the brain of fed mice but was lower or unchanged in the hypothalamus and adipose tissue. *Kctd15* expression was upregulated in the hypothalamus, brain and adipose tissue of fed mice and downregulated by high fat feeding in liver, adipose tissue and the hypothalamus but not the remainder of the brain. *Sh2b1* expression in the brain and *Faim2* expression in adipose tissue were specifically increased >20-fold in fed mice. *Tmem18* expression in adipose tissue but not the brain was reduced 80% by high fat feeding. Few changes in the expression of these genes were observed in liver.

**Conclusions:**

These data show nutritional regulation of nearly all these GWAS obesity genes, particularly in the brain and adipose tissue, and provide support for their role in the development of obesity. The complex patterns of nutritional and tissue-dependent regulation also highlight the difficulty that may be encountered in determining how the GWAS genetic variants affect gene expression and consequent obesity risk in humans where access to tissues is constrained.

## Background

Advances in our understanding of the genetic contribution to obesity have come from recent genome wide association studies (GWAS). GWAS have identified several new genetic variants associated with obesity and its related traits such as body mass index (BMI). Most prominent amongst these are SNPs within *FTO*, which have the strongest effects on obesity risk [1-5]. Additional studies, including large scale meta-analyses, discovered additional variants associated with obesity-related traits that have now been replicated [5-7]. These include SNPs within or near *MC4R, CTNNBL1*, *NPC1*, *MAF*, *PTER*, *PRL*, *SH2B1*, *NEGR1*, the *SEC16B-RASAL2* region, *TMEM18*, the *SFRS10-ETV5-DGKG* region, *BDNF*, *FAIM2*, *KCTD15*, the *NCR3AIF1BAT2* region, *GNPDA2*, and *MTCH2*[8-11].

Alterations in a few of these genes have previously been shown to affect body weight [12-16]. Melanocortin 4 receptor, *MC4R,* mutations are the most common cause of monogenic severe obesity and missense variants within the gene have been associated with altered risk for common obesity [16,17]. Brain derived neurotrophic factor, *BDNF,* regulates feeding, its expression is altered by consumption of high fat diet, and variants within the gene have previously been associated with obesity [13,14,16,18]. *Sh2b1* knockouts are obese, and this gene is known to play important roles in leptin signalling [15,19]. Understanding the site of action and function of these new obesity genes may provide valuable insight into the pathogenesis of obesity. However, despite the extensive investments in GWAS studies, to date studies of the biology of these genes have been limited. Little is known about most of these GWAS obesity genes and their role in the regulation of body weight is unknown. A better understanding of these genes is needed to capitalize on the information generated by the GWAS.

The SNPs identified by the GWAS generally do not affect the amino acid sequence of the mature protein. They are typically intronic or intergenic. The location of these SNPs suggests they likely affect the regulation of the gene they are located within or of nearby genes, and in some cases it is not clear which of the nearby genes they may be affecting [7,8,11,20]. Regulation of a gene may also be tissue specific, or occur in response to certain physiological states. Many genes involved in the regulation of energy homeostasis are metabolically regulated [21-24]. Expression of *FTO* was shown to be regulated by feeding and fasting in the hypothalamus [25]. Thus we hypothesize that if these GWAS genes affect the development of obesity they may be nutritionally regulated.

Knowledge of the regulation of the GWAS obesity genes is critical for understanding how the associated SNPs alter the function of their cognate genes and thus how these genes may affect the development of obesity. This requires knowledge of the tissues where the gene is normally expressed and an understanding of the physiological processes that regulate it. We sought to determine whether metabolic factors affect the regulation of the newly discovered obesity genes. We examined the regulation of these genes by feeding/fasting status and by the consumption of a diet high in fat and sugar. Here we show that most of the recently identified obesity genes are regulated by dietary status, providing support for their role in processes relevant to the development of obesity. These patterns of regulation were often tissue-dependent and largely unique for each gene, suggesting that many of these genes affect distinct pathways in the development of obesity and that analysis of the effects of the GWAS SNPs on gene expression may need to be performed in all physiologically relevant tissues and under multiple physiological contexts.

## Methods

### Animals

Animals were housed in an environmentally controlled facility with 14 hour light cycles (7 am – 9 pm). Female C57BL/6 J mice were given free access to water and either a standard rodent chow (LabDiet 5010, PMI Nutrition, St. Louis MO, USA) or a diet containing 60% calories from fat (primarily lard) and 20% calories from sugar (sucrose and maltodextrin; D12492, Research Diets, New Brunswick NJ, USA) from weaning. This high fat diet has a cholesterol content of 300.8 mg/kg from the lard. The mice were sacrificed by CO_2_ asphyxiation at 8 weeks of age. Tissues were rapidly dissected and immediately placed in liquid nitrogen then stored at −80°C. Prior to sacrifice, animals were either fasted 4 hours (9 am – 1 pm) or dissections were performed at 9 am without prior removal of food (“fed”). All procedures were approved by the UBC Committee on Animal Care and were performed according to Canadian Council on Animal Care guidelines.

### RNA extraction and cDNA synthesis

Prior to extraction, tissue samples were cut into small pieces and placed in RNA Later Ice (Applied Biosystems, Carlsbad CA, USA) at −20°C for at least 24 hrs. RNA was extracted using a commercially available kit (E.Z.N.A.™ Total RNA II, Omega Biotek, Norcross GA, USA). The integrity of the RNA was verified by performing formaldehyde denaturing agarose gel electrophoresis for all samples except the hypothalamus, for which too little RNA was obtained. Following DNAse I digestion (Fermentas, Burlington ON, Canada) to remove any contaminating genomic DNA, cDNA synthesis (RevertAid First Strand cDNA Synthesis Kit, Fermentas, Burlington ON, Canada) was performed using 1 μg of RNA with mixed oligo-dT and hexamer primers (20 ng/μL oligo-dT, 3.6 ng/μL random hexamer final concentration) according to the manufacturer’s directions. RNA was subsequently removed with RNase H digestion (Fermentas, Burlington ON, Canada). Successful cDNA synthesis was verified by PCR amplification of our control genes and visualization of a band of the appropriate size on an agarose gel.

### Gene expression

We examined the dietary regulation of *Aif1*, *Bat2*, *C10orf97*, *Ctnnbl1*, *Dgkg*, *Etv5*, *Faim2*, *Gnpda2*, *Kctd15*, *Maf*, *Mtch2*, *Negr1*, *Npc1*, *Pter*, *Rasal2*, *Sec16b*, *Sfrs10*, *Sh2b1*, and *Tmem18*. Primer sequences are provided in Additional file 1 Table S1. When there were possible alternatively spliced forms identified in the UCSC genome browser ( http://www.genome.ucsc.edu), we designed the primers in regions common to all isoforms. All primer pairs spanned an intron. *Ncr3* is a pseudogene in mice. It does not encode a functional protein due to the presence of premature stop codons [26] and was not examined in our studies.

Relative gene expression was measured using a StepOne Plus real time thermocycler (Applied Biosystems Inc., Carlsbad CA, USA), using SYBR green I for detection (PerfeCTa SYBR Green FastMix, VWR International, Edmonton AB, Canada). Forty cycles of amplification were performed followed by melt-curve analysis for verification that only a single product was amplified from each sample. This verification was also performed by direct visualization of PCR amplification products on an agarose gel. Baseline correction, threshold setting, and calculation of the Ct value were performed automatically by the StepOne software (version 2.1, Applied Biosystems, Carlsbad CA, USA).

Cyclophilin (*Ppib*) was chosen as the reference gene, as it proved most robust to changes in expression by experimental condition across each of the tissues in comparison to beta-actin (*Actb*), *Gapdh*, and *Arbp*. This control gene was included on several plates contemporaneous with the genes being assessed, and for each sample the average value of this gene across its multiple replicates was used for normalization of that sample. Delta Ct (ΔCt) values were calculated by subtracting the cycle threshold (Ct) value for each gene from this average value. Delta delta Ct (ΔΔCt) values were calculated for each sample by subtracting its ΔCt value from the average ΔCt for the chow-fed, fasted controls in the same experiment. Initially, single measurements were made for each sample (n = 5 per dietary group). When there was a potential difference between groups, replicate experiments were performed, including additional samples (up to 10 animals per group). The numbers vary for each tissue and gene because of poor-quality or low yields of RNA obtained from some samples and the number of samples with sufficient RNA available at the time for analysis. For each sample, the average ΔΔCt of all its replicates across experiments was used for statistical analysis.

### Statistical analysis

Our study was comprised of three groups: chow-fed mice fasted prior to tissue collection (controls); chow-fed mice not fasted prior to tissue collection, “fed”; and high fat diet-fed mice that were fasted prior to tissue collection. The average ΔΔCt values of the high fat-fed and fed groups were compared to that of the fasted chow-fed control group and the significance of each assessed by a non-parametric Mann–Whitney U test because of the small sample size. Because this results in two comparisons for each gene (chow-fed, fasted vs. non-fasted mice; fasted chow-fed vs. fasted high fat diet-fed mice), the resulting P-values were subsequently Bonferroni corrected by multiplying them by 2. This approach was chosen over a Kruskal-Wallis test because the comparison between chow-fed “fed” mice and fasted high fat diet-fed mice that is accounted for in the Kruskal-Wallis test is not meaningful. The average fold change for each group shown in the figures was calculated as 2^-average ΔΔCt^, with the error bars as 2^-average ΔΔCt ± its SE^[27].

## Results

### Regulation of GWAS obesity genes by dietary status in hypothalamus

**Figure 1:**
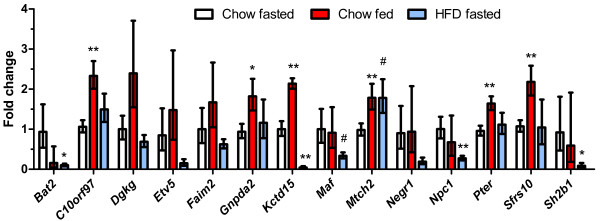
**Regulation of GWAS obesity genes in the hypothalamus in the fed state and by high fat feeding.** Open bars represent the chow-fed, fasted controls; red bars represent the chow-fed non-fasted group; and blue bars represent the high fat fed group (fasted prior to tissue collection). The number of mice in the chow fasted, chow fed, and HFD fasted groups were: *Bat2* (10, 5, 7), *C10orf97* (10, 7, 5), *Dgkg* (4, 5, 5), *Etv5* (10, 7, 7) *Faim2* (4, 5, 5), *Gnpda2* (9, 9, 6), *Kctd15* (7, 5, 6), *Maf* (7, 7, 7), *Mtch2* (10, 9, 7), *Negr1* (10, 7, 7), *Npc1* (10, 5, 7), *Pter* (10, 8, 6), *Sfrs10* (10, 7, 5) and *Sh2b1* (9, 6, 7), respectively. P-values vs. the chow-fed fasted controls: * <0.05, ** <0.01, ^*#*^ <0.1.

All 19 of the GWAS genes we examined were expressed in the hypothalamus. Many were regulated by either feeding/fasting or by consumption of the high fat diet (Figure 1), but only two genes, *Kctd15* and likely *Mtch2*, were regulated by both conditions. The expression of many genes was increased in non-fasted animals. *Kctd15* expression was increased 2.1-fold in fed animals, *C10orf97* expression was increased 2.3-fold, and expression of *Sfrs10* was also increased an average of 2.2-fold. The expression of *Pter* was increased 1.6-fold, and we detected a 1.8-fold increase in *Gnpda2* expression. In addition, *Ctnnbl1* expression was increased an average of 1.5-fold, although this did not reach statistical significance. Similarly, the expression of *Mtch2*, *Faim2* and *Dgkg* were also increased in the fed mice compared to fasted, however due to the large variability in expression levels, these were not significant.

In contrast to the small differences in gene expression observed in fed compared to fasted animals, chronic feeding of a high fat diet resulted in substantial changes in expression (Figure 1). *Kctd15* was decreased a marked 20-fold in animals consuming a high fat diet (i.e. expression was ~5% of that seen in chow-fed, fasted control mice). *Bat2* and *Sh2b1* mRNA levels were decreased ~10-fold in animals fed the high fat diet. *Npc1* gene expression was decreased by ~4-fold. *Maf* expression was ~3-fold lower, but due to the high variability in the control group did not quite statistical significance after Bonferroni correction. Expression of *Etv5* and *Negr1* was only 15% and 19%, respectively, of that observed in controls, although neither reached statistical significance. *Mtch2* was the only gene that had increased expression with high fat feeding in the hypothalamus. We did not observe any significant changes in hypothalamic gene expression with feeding or consumption of a high fat diet for *Aif1*, *Dgkg*, *Faim2*, *Rasal2*, *Sec16b* or *Tmem18* (not shown).

### Regulation of GWAS genes by dietary status in whole brain excluding hypothalamus

**Figure 2:**
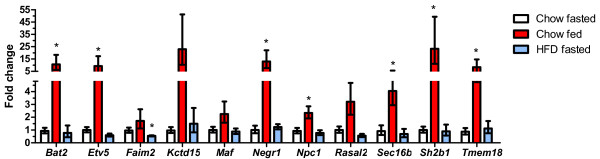
**Regulation of GWAS obesity genes in the remainder of the brain in the fed state and by high fat feeding.** Open bars represent the chow-fed, fasted controls; red bars represent the chow-fed non-fasted group; and blue bars represent the high fat fed group (fasted prior to tissue collection). The number of mice in the chow fasted, chow fed, and HFD fasted groups were: *Bat2* (7, 10, 5), *Etv5* (7, 10, 7) *Faim2* (7, 10, 7), *Kctd15* (5, 10, 5), *Maf* (7, 10, 7), *Negr1* (7, 10, 5), *Npc1* (7, 10, 5), *Rasal2* (7, 10, 6), *Sec16b* (7, 10, 5), *Sh2b1* (7, 10, 7), and *Tmem18* (7, 10, 5), respectively. *P <0.05 vs. the chow-fed fasted controls.

Many regions of the brain outside the hypothalamus are also important for the regulation of food intake and energy balance [28]. We detected expression of all 19 genes in these other regions of the brain. Similar to the hypothalamus, the expression of many genes differed between the fasted and fed groups (Figure 2). In contrast to the hypothalamus, however, the changes in expression were often much larger and occurred primarily in non-fasted animals. Most notably, expression of *Kctd15* and *Sh2b1* were increased >20-fold, and expression levels of *Bat2, Etv5, Negr1*and *Tmem18* were increased ~10-fold in the fed group. Changes in *Kctd15* expression likely did not reach statistical significance due to a large variability in the increase in expression. While the magnitude was variable, most samples had at least 15-fold increased *Kctd15* expression. Feeding was also associated with a 2 to 4 fold increase in the expression of *Sec16b*, *Rasal2*, and *Npc1*. We observed similar trends in the expression of *Maf* that did not reach statistical significance.

In marked contrast to what was observed in the hypothalamus where several genes were substantially downregulated by high fat feeding, the expression of only a single gene in the remainder of the brain was altered by high fat feeding. Expression of *Faim2* was reduced ~50% in the high fat-fed group compared to the chow-fed mice (Figure 2). We did not observe any differences in the expression of *Aif1*, *Mtch2*, *Dgkg*, *Sfrs10*, *Pter*, *Ctnnbl1*, *Gnpda2* or *C10orf97* under any condition in the remainder of the brain (not shown).

### Regulation of GWAS genes by dietary status in adipose tissue

**Figure 3:**
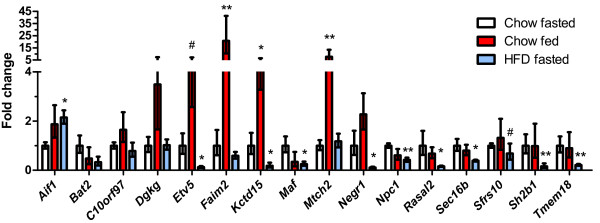
**Regulation of GWAS obesity genes in adipose tissue in the fed state and by high fat feeding.** Open bars represent the chow-fed, fasted controls; red bars represent the chow-fed non-fasted group; and blue bars represent the high fat fed group (fasted prior to tissue collection). The number of mice in the chow fasted, chow fed, and HFD fasted groups were: *Aif1* (7, 8, 9), *Bat2* (7, 6, 9), *C10orf97* (7, 8, 5), *Dgkg* (7, 6, 4), *Etv5* (7, 4, 8), *Faim2* (7, 6, 7), *Kctd15* (7, 4, 9), *Maf* (7, 7, 9), *Mtch2* (7, 8, 5), *Negr1* (7, 4, 8), *Npc1* (7, 8, 9), *Rasal2* (7, 7, 9), *Sec16b* (7, 5, 8), *Sfrs10* (7, 8, 5), *Sh2b1* (7, 5, 9), and *Tmem18* (7, 6, 9), respectively. P-values vs. the chow-fed fasted controls: * <0.05, ** <0.01, ^*#*^ <0.1.

All the GWAS genes we assessed were also expressed in white adipose tissue. Several GWAS obesity genes were highly differentially regulated in adipose tissue from high fat-fed compared to chow-fed mice. Many genes had reduced expression in high fat diet-fed mice (Figure 3). Most strikingly, we observed a ~10-fold reduction in expression of *Negr1* in the high fat-fed mice compared to the chow-fed controls. *Etv5* and *Kctd15* were also markedly downregulated in mice consuming the high fat diet (~8-fold and 5-fold, respectively). Decreased expression of *Maf*, *Npc1*, *Rasal2*, *Sec16b*, *Sh2b1*, and *Tmem18* in high fat diet-fed mice ranged from ~2.5-fold for *Npc1* to 6-fold for *Rasal2*. We observed a similar decrease in *Bat2* expression with high fat feeding (3-fold), although this did not reach statistical significance. In contrast, *Aif1* had a small but significant increase in gene expression in adipose tissue of the mice fed a high fat diet (2.1-fold).

We found the expression of three genes changed significantly by feeding status in adipose tissue. *Faim2* was upregulated a remarkable 20-fold in fed versus fasted mice consuming normal chow (Figure 3). Expression of *Mtch2* was increased an average of 8-fold and *Kctd15* was increased ~5-fold in fed animals. A similar trend was observed for *Etv5*, which had 4-fold higher expression in adipose tissue of fed mice. Smaller increases were observed for *Dgkg* and *Negr1* (3.5 and 2.3-fold, respectively), but these were not significant. We did not detect any significant differences in gene expression for *Dgkg*, *Ctnnbl1*, *Sfrs10*, *Bat2, Pter*, *Gnpda2*, or *C10orf97* in either group.

### Regulation by dietary status in liver

**Figure 4:**
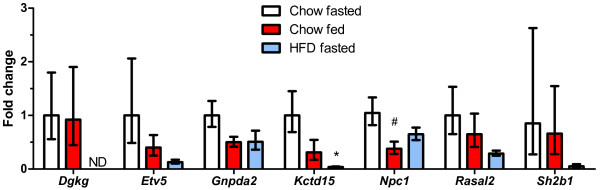
**Regulation of GWAS obesity genes in the liver in the fed state and by high fat feeding.** Open bars represent the chow-fed, fasted controls; red bars represent the chow-fed non-fasted group; and blue bars represent the high fat fed group (fasted prior to tissue collection). The number of mice in the chow fasted, chow fed, and HFD fasted groups were: *Dgkg* (5, 4, 6), *Etv5* (7, 5, 3), *Gnpda2* (7, 5, 6), *Kctd15* (5, 4, 4), *Npc1* (7, 5, 5), *Rasal2* (7, 5, 6), and *Sh2b1* (7, 5, 6), respectively. P-values vs. the chow-fed fasted controls: * <0.05, ^*#*^ <0.1. ND = not detected.

Of the 19 genes we assessed, all except *Faim2* and *Negr1* were expressed in the liver. In contrast to the tissues above, we observed few differences in gene expression in the liver (Figure 4). Expression of *Kctd15* was decreased an average of 20-fold in high fat diet-fed mice compared to chow-fed mice, and was the only gene for which significant differences were detected. *Sh2b1* expression was decreased by a similar amount but was not significant (P = 0.14) likely due to the variability of the controls. *Rasal2* expression was decreased an average 3.4-fold in the high fat diet-fed mice. Expression of *Gnpda2* was decreased 2-fold in the high fat-fed mice, and by a similar amount in fed animals compared to fasted. Similarly, *Npc1* expression was decreased an average 2.6-fold in fed animals compared with fasted. Interestingly, expression of *Dgkg* was low but detectable in both groups of chow-fed mice, whereas expression could not be detected in five of six high fat diet-fed mice. No differences were observed in the expression of the other genes expressed in liver (*Sec16b*, *Maf*, *Mtch2*, *Tmem18*, *Aif1*, *Bat2*, *Sfrs10*, *Pter*, *Ctnnbl1*, *Etv5*, and *C10orf97*) in response to fed status or consumption of the high fat diet.

## Discussion

GWAS have identified many new SNPs associated with the development of obesity. Most of these SNPs are located in introns or intergenic regions, suggesting they affect the regulation of the corresponding gene or nearby gene(s), but which gene or genes they affect is unknown. Little is known about most of these genes or how they may affect the development of obesity, but understanding their regulation is a key step that may provide important clues. Many genes involved in metabolism and maintaining energy balance are regulated in response to feeding and fasting or by dietary components. Identification of such regulatory patterns would provide support for a potential role in the development of obesity. To begin to understand the regulation of the GWAS obesity genes and if they are nutritionally regulated we examined how changes in dietary conditions affect their expression.

We observed complex, tissue-dependent regulation of most of these new obesity genes. Only one gene, diacylglceryol kinase gamma (*Dgkg*), did not significantly change under any condition tested. However, given the role of this kinase in second messenger signalling, it is likely that it may not be physiologically regulated at the level of transcription but rather by activity of the enzyme. Note that this does not preclude the possibility that the GWAS SNPs may affect its basal transcription levels. The regulation of some genes was highly tissue specific. Four transcripts, *Sfrs10*, *C10orf97*, *Pter*, and *Ctnnbl1* were increased in hypothalami of fed animals compared to fasted, but were not changed by this condition in other regions of the brain, liver or adipose tissue, or by high fat feeding in any tissue. Thus these genes may have unique functions in the hypothalamus regulating energy balance and the development of obesity. Similarly, *Aif1* (allograft inflammatory factor 1) expression was increased by high fat feeding only in adipose tissue, whereas no differences in its expression were observed in the other tissues or in fed animals. It is unclear whether this reflects increased inflammation of adipose tissue in the high fat-fed mice (e.g. expression from increased amounts of inflammatory cells in the tissue), or whether this suggests that the role of *Aif1* in obesity may be mediated via specific actions in adipose tissue.

In contrast to the above genes whose expression was affected by a single condition in a single tissue, the expression of other genes was more complex and altered in multiple tissues or by both conditions. *Gnpda2* expression was increased in the hypothalamus and decreased in the liver of fed animals, whereas its expression did not differ in other regions of the brain or adipose tissue in fed vs. fasted animals. We did not detect any significant changes of this gene in response to high fat diet consumption, although another group has recently reported decreased expression of this gene in high fat diet-fed rats [29]. Several transcripts were decreased in the hypothalamus by high fat feeding and increased in the remainder of the brain in the fed state, with variable patterns in adipose tissue. *Kctd15* regulation was the most consistent across tissues, with its expression being increased with feeding in hypothalamus, brain and adipose tissue (but not liver) and decreased by high fat diet consumption in hypothalamus, adipose tissue and liver (but not brain). A recent study has also found decreased *Kctd15* expression in hypothalamus and adipose tissue of high fat-fed rats, although they did not observe reduced expression in liver [29].

We also observed a large range in the magnitude of the changes in gene expression, and for each transcript the magnitude of the change in expression was not the same in each tissue. For example, expression of *Kctd15* was increased >20-fold in the brain of fed animals, but only by 2- to 4-fold in the hypothalamus and adipose tissue. These complex and tissue-dependent patterns of regulation in response to nutritional status suggest that these genes have important and perhaps distinct roles in several tissues.

Although each gene showed distinct patterns and magnitude of regulation across tissues and nutritional conditions, we observed striking consistency in the overall pattern of regulation by feeding and chronic high fat diet consumption. With few exceptions (*Npc1* and *Gnpda2* in liver, Figure 4), all genes regulated by feeding/fasting were upregulated in the non-fasted animals. This suggests that most transcripts respond to nutrient intake or increased nutrient levels, rather than acting as cues initiating food intake or promoting the mobilization of energy stores. Several transcription factors, co-activators, and factors affecting chromatin structure are known to increase expression of target genes in response to changes in cellular nutrient levels and energy status and thus may be important for the regulation of these genes [21-23]. Regulation of gene expression by feeding was prominent in the hypothalamus and remainder of brain, and also for some transcripts in adipose tissue. In fact, changes in response to feeding-fasting status accounted for nearly all the expression differences we observed in the brain; only one gene (*Faim2*) was regulated by high fat feeding in the brain. Interestingly, however, there was little overlap in the genes regulated by feeding between the hypothalamus and the remainder of the brain, suggesting that distinct nutrient-responsive regulatory pathways operate in various regions of the brain. In contrast, consumption of a high fat diet was associated with a reduction in expression of all of the regulated transcripts with the exception of *Mtch2* in hypothalamus and *Aif1* in adipose tissue. High fat diet consumption affected gene expression mainly in adipose tissue and the hypothalamus but not the rest of the brain, suggesting energy storage and homeostatic regulation of energy balance may be preferentially affected by today’s obesogenic diets. This also suggests that high fat diets may predominantly down-regulate pathways important for normal adipose tissue function. Previous studies have shown reduced adipogenic gene expression in obese individuals [30-32].

Our findings further suggest that the effects of these genes on the development of obesity may occur in tissues in addition to the brain, which was previously suggested to be their likely site of action [8,11]. Consistent with this, *Negr1* has recently been shown to be expressed in adipose tissue and to be associated with gene expression networks [33]. *Mtch2* expression in adipose tissue is increased in obese individuals [34] and has been shown to be increased in high fat-fed rats [29], although we did not observe changes in its expression in adipose tissue in response to high fat feeding (Figure 3). Somewhat surprisingly given its key role in whole-body metabolism and that high fat diets have previously been shown to regulate the hepatic expression of many genes [32], very few of the GWAS obesity genes had altered expression in the liver, suggesting that the metabolic processes of the liver may not play a significant role in the maintenance of whole body energy homeostasis. We also found that most of these genes except *Etv5, Mtch2, Pter* and *Tmem18* had relatively low to no detectable expression in soleus muscle (not shown). A recent study examining the expression of a partially-overlapping subset of GWAS obesity genes also found modest changes in expression of only a few genes in liver and muscle [29].

The nutritional regulation of these new obesity genes provides support for their potential role in the regulation of energy balance. These data have several important implications for studies examining the effects of the GWAS SNPs on gene regulation and ultimately the mechanisms by which these genes affect the development of obesity. The highly tissue-dependent patterns of regulation suggest that all tissues where a gene is expressed will need to be examined to determine whether the particular SNPs identified by the GWAS affect the expression of a gene. This is further highlighted by the complex patterns and variability in magnitude of expression changes for a transcript in different tissues. Analysis of the effects of the GWAS SNPs on gene expression in lymphocytes has been suggested since this is a readily accessible source of RNA, and differences in lymphocyte gene expression between lean and obese individuals have been detected [35]. This may be useful if the SNPs affect basal transcription of a gene, but will not be useful if the SNPs affect enhancer elements. Indeed, in a study examining the effects of diabetes-associated SNPs on gene expression, many correlations between genotype and expression were found in muscle and adipose tissue that were not observed in lymphocytes [36]. Similarly, SNPs affecting gene expression in lymphocytes were only associated with inflammatory and auto-immune diseases not with metabolic diseases [37]. Thus it may be very difficult to pinpoint the effects of these obesity GWAS SNPs on transcript levels. To understand the role of these genes in the development of obesity it will be necessary to fully understand the role of each gene in each physiologically relevant tissue, as they are regulated and thus may behave differently depending upon the tissue being examined.

Transcriptional regulatory elements can be located before, within, or even following a gene, and can be located at substantial distance (up to 1 Mb) from the gene [38]. Thus it is possible that the variants detected by the GWAS studies affect the regulation of other nearby genes, not just those that are closest to the SNP. We chose to focus our analyses on the genes reported by the GWAS studies as the most likely genes affected by the associated variants. The functions of many of these genes are not well described, and it is not clear how they may affect the development of obesity. We performed these studies to learn more about these genes, determine whether their expression patterns were consistent with them having a role in the regulation of body weight, and examine whether their expression is affected by known metabolic regulatory factors. While our data provide support for all the genes having a potential role in the development of obesity, they do not confirm that these are “the genes” nor do they exclude the possibility that the GWAS SNPs affect the regulation of other nearby genes.

Three of the obesity SNPs identified by Thorliefsson and colleagues [8] were found in gene dense regions where the SNP was not clearly more closely related to a single gene, and were consequently identified as being within gene clusters: *SEC16BRASAL2*, *SFRS10ETV5DGKG*, and *NCR3AIF1BAT2.* We examined the expression of the genes in each cluster with the hope of discovering regulatory patterns that may point to the more likely causative gene in each cluster. Unfortunately these studies provide little support favoring one of the genes over the other(s). All the genes were expressed in tissues relevant to the regulation of body weight. For the first cluster, both *Sec16b* and *Rasal2* were increased in the fed state in the brain and decreased by high fat feeding in adipose tissue. Neither was altered in the hypothalamus, while *Rasal2* expression was also decreased in response to high fat feeding in the liver. The similar expression patterns make it difficult to prioritize one over the other as likely being the causative gene and also suggest that there may in fact be common regulatory elements affecting multiple genes in the region. The genes in the second and third clusters showed very different expression patterns from each other. In the second cluster, *Sfrs10* expression was increased in the hypothalamus in fed mice but did not change in any of the other tissues. *Etv5* expression in the hypothalamus was decreased by high fat feeding and its expression increased in the brain and adipose tissue of fed animals. *Dgkg* expression, although relatively high in the hypothalamus and remainder of the brain, was not affected by fasting or the consumption of a high fat diet. For the third cluster, differential expression of *Aif1* was detected in adipose tissue, where it was the only gene to increase in response to a high fat diet. In contrast, *Bat2* expression was decreased by the high fat diet in hypothalamus, increased by feeding in brain, and was not changed in liver or adipose tissue. The expression of the *Ncr3* pseudogene was not analyzed. Thus each member of the last two groups has a differential regulation in response to changing dietary conditions, which suggests that the causative variant(s) at these loci may affect just one of the genes rather than the coordinate regulation of multiple genes. However, for each cluster all the genes had potentially relevant changes in expression and it is unclear which of the genes is most likely to be affected by the GWAS SNP based on these analyses.

Recently other studies have reported expression patterns of some of these genes. One of these has shown that *Tmem18* is expressed in most neurons [39]. These studies found no change in its expression in the hypothalamus or brainstem with prolonged (16 or 24 hour) fasting, 48 hour sucrose or fat ingestion or in response to chronic addition of 10% sucrose to the diet compared to ad-lib chow-fed mice [39]. Consistent with these findings, we saw no evidence of altered *Tmem18* expression in response to chronic consumption of a high fat-sucrose diet. In contrast, however, we found ~8-fold higher levels of *Tmem18* in the brain of non-fasted animals (Figure 2). A recent study reproduced our finding of reduced expression of *Etv5* and *Sh2b1* in the hypothalamus and *Kctd15* in the hypothalamus and adipose tissue of high fat-fed animals [29]. In contrast, this study also found decreased expression of *Kctd15* and *Gnpda2* in the hypothalamus and *Tmem18* in the liver, hypothalamus and soleus muscle, and increased expression of *Mtch2* in adipose tissue and *Pter* in adipose tissue and liver of high fat-fed rats, which we did not observe [29]*.* The reasons for these discrepancies may relate to experimental design, such as differences in the species and strain studied; the diet used and its duration; the time of day of sacrifice; and the fasting conditions used, and require further investigation.

## Conclusions

These studies have shown that the regulation of these new GWAS obesity genes in response to dietary conditions is complex and often tissue-dependent. Knowledge of the regulation of these genes will help in determining their role in obesity. These data provide supportive evidence for a role of these genes in the regulation of energy metabolism, but suggest that discovery of the mechanism by which the regulatory SNPs identified in the GWAS affect these genes will require tissue and context specific analysis.

## Abbreviations

*Aif1*: Allograft inflammatory factor 1; BMI: Body mass index; *Ctnnbl1*: Catenin, beta-like 1; *C10orf97*: Chromosome 10 open reading frame 97 a; *Fam188a*: Which is now known as Family with sequence similarity 188 member A; Ct: Cycle threshold; *Dgkg*: Diacylglycerol kinase gamma; *Etv5*: Ets variant 5; Faim2: Fas apoptotic inhibitory molecule 2; GWAS: Genome-wide association study; *Gnpda2*: Glucosamine 6 phosphate deaminase 2; HFD: High fat diet; *Bat2*: HLA-B associated transcript 2; Mb: Million base pairs; *Mtch2*: Mitochondrial carrier homolog 2; *Negr1*: Neuronal growth regulator 1; *Npc1*: Niemann Pick type C1; *Pter*: Phosphotriesterase related; *Kctd15*: Potassium channel tetramerisation domain containing 15; *Rasal2*: Ras protein activator like 2; *Sec16b*: SEC16 homolog b from S. cerevisiae; SNP: Single nucleotide polymorphism; qPCR: Real-time quantitative reverse transcription PCR; *Tra2b* originally named *Sfrs10*: Transformer 2 beta homolog; *Sh2b1*: Sh2b adaptor protein 1; *Tmem18*: Transmembrane protein 18; *Maf*: v-maf musculoaponeurotic fibrosarcoma oncogene homolog.

## Competing interests

The authors confirm that they have no competing interests to disclose.

## Authors' contributions

PY, SK and MMH conducted the study and participated in writing the manuscript. SMC designed the study, analyzed and interpreted the results and wrote the manuscript. All authors read and approved the final manuscript.

## Supplementary Material

Additional file 1 **Table S1.** Primer sequences used in these studies. Click here for file
